# Host‐Associated Differentiation in a Parasitoid Wasp Connected to Venom‐Related but Not Chemosensory Genes

**DOI:** 10.1111/mec.70589

**Published:** 2026-10-12

**Authors:** Xuyue Yang, Marco Fracassetti, Tanja Slotte, Christopher W. Wheat, Peter A. Hambäck

**Affiliations:** ^1^ Department of Ecology, Environment and Plant Sciences Stockholm University Stockholm Sweden; ^2^ Science for Life Laboratory (SciLifeLab) Stockholm University Stockholm Sweden; ^3^ Department of Zoology Stockholm University Stockholm Sweden

## Abstract

The mechanisms driving the megadiversity of parasitic wasps remain a central question in evolutionary ecology. To investigate the genomic architecture underlying host‐associated differentiation in parasitic wasps, we generated a *de novo* genome assembly and performed whole‐genome re‐sequencing of 17 populations of the parasitoid wasp *Asecodes parviclava* (Eulophidae) across a latitudinal gradient and multiple *Galerucella* host species. Despite panmixia in host populations, we identified three genetically differentiated wasp lineages, with the most profound divergence separating *Galerucella tenella*‐associated wasps from other populations. Locus‐specific scans revealed that selection acts highly asymmetrically. We detected significant differentiation and copy‐number expansion clustered within venom‐related gene families, contrasting sharply with a near‐complete lack of divergence in chemosensory genes. This decoupled evolution suggests that host finding may rely on behavioural plasticity, whereas physiological compatibility is genetically hard‐wired via multi‐layered immune‐suppression mechanisms. Finally, asymmetrical gene flow in northern populations demonstrates that fine‐scale habitat overlap creates ecological connectivity without eroding specialized lineages, as intense host‐mediated selection purges maladapted hybrids. Ultimately, our study demonstrates that physiological barriers, rather than behavioural recognition, may maintain specialized parasitoid lineages, providing new insights into the genomic basis of speciation with gene flow in specialized natural enemies.

## 1 Introduction

The staggering megadiversity of parasitic wasps (Forbes et al. 2018) presents a fundamental question in evolutionary biology: what mechanisms drive such rapid and pervasive speciation? While intrinsic factors like haplodiploidy are thought to accelerate the fixation of adaptive alleles in wasps (Bendall et al. 2022), the key extrinsic driver is almost certainly the complex multidimensional niche provided by the hosts. This diversification process commonly unfolds via host‐associated differentiation, where populations of polyphagous or oligophagous consumers adapt to distinct host species (Forbes et al. 2017). Because host‐use landscapes are dynamic, range shifts can expose parasitoids to novel species networks over time, continually generating new opportunities for population isolation and further diversification (Hambäck et al. 2024).

An expected driver of this diversification is the coevolutionary ‘arms race’ between hosts and parasitoids, where hosts evolve sophisticated defences and wasps evolve mechanisms to overcome them, causing interacting sets of populations to evolve away from other interacting sets through time. Since hosts vary in their defensive weaponry, wasps frequently face trade‐offs in their ability to utilize different species, further promoting differentiation and specialization (Antolin et al. 2006; Dubuffet et al. 2007). This dynamic is particularly intense in endoparasitic wasps, whose larvae must develop inside the host—an environment that is simultaneously a resource and a hostile environment because of immune defences. Unravelling this dynamic interaction is pivotal for deciphering the genomic mechanisms underlying parasitic wasp megadiversity.

The primary host defence against endoparasitoids is a cellular immune reaction in which hemocytes encapsulate the wasp egg, ultimately killing the egg via asphyxiation. This high stakes survival game imposes strong selection on wasps to suppress host defences (Alvarado et al. 2020), which in turn drives counter‐adaptation in the host (Kraaijeveld et al. 1998; Wertheim 2022). Interestingly, the pace of this coevolutionary arms race may be asymmetrical, as parasitoids frequently adapt to host defences faster than hosts can evolve resistance to parasitoid virulence (Hambäck and Janz 2026). This evolutionary asymmetry is supported by experimental evolution studies accordingly showing rapid wasp adaptation within just a few generations (Cavigliasso et al. 2019), as well as cross‐infection experiments demonstrating stronger local adaptation in parasitic wasps compared to their host (Dupas et al. 2003; Phillips et al. 2002).

Previous comparative studies of parasitoids utilizing sympatric hosts often reveal pronounced interspecific differences in attack strategies and success rates. For instance, the distinct *Drosophila*‐parasitizing genera *Leptopilina*, *Ganaspis* and *Asobara* target entirely different components of the host immune cascade (e.g., Huang et al. 2021; Mortimer 2013; Prevost et al. 2005; Rizki et al. 1990; Schlenke et al. 2007). In contrast, investigations into intraspecific variation in virulence remain sparse and are heavily skewed toward *Drosophila* laboratory models (Dupas et al. 2009; Kimura and Novkovic 2015; Kraaijeveld and Godfray 1999). While non‐model systems have occasionally been examined, they typically focus on generalized virulence or intra‐population polymorphisms rather than spatial and macro‐geographic variation (Dion et al. 2011; Henter 1995; Kaiser et al. 2015; Zhang et al. 2021). Our previous phenotypic work addressed this empirical gap by demonstrating that parasitism rates in *Asecodes parviclava* depend interactively on both the specific *Galerucella* (Coleoptera: Chrysomelidae) host species and the geographic origin of the wasp population (Fors et al. 2016). In this paper, we leverage whole‐genome data to dissect the genomic architecture underlying these phenotypic shifts, shifting the focus from macro‐geographic variance to the precise candidate loci driving population‐level divergence in virulence.

The evolutionary trajectories of diverging parasitoid populations are shaped by two tightly coupled ecological requirements: the behavioural capacity to locate and identify a specific host and the physiological capacity to overcome its unique immune defences. Consequently, host‐associated differentiation is theoretically expected to target two primary functional toolkits in tandem: chemosensory genes regulating host recognition and venom genes regulating immunosuppression (Martinson et al. 2017). Venom proteins, synthesized in the female venom gland, are indispensable functional tools for neutralizing host counter‐resistance (Alvarado et al. 2020; Price et al. 2009; Xin et al. 2017). Beyond disrupting hemocyte behaviour or inducing cell lysis (Lin et al. 2019; Wang et al. 2013), venom can execute sophisticated intracellular overhauls, including the epigenetic silencing or post‐transcriptional decay of host immune transcripts. Parallel to this physiological weaponry, host‐associated divergence could be expected to target the chemosensory apparatus—primarily comprised of odorant (ORs), gustatory (GRs) and ionotropic (IRs) receptors—which allows parasitoids to navigate complex chemical landscapes (Ahmed et al. 2016; Robertson et al. 2010; Sheng et al. 2017). Because a mismatch between host recognition and immune suppression could be evolutionarily fatal, we may expect that these sensory and virulence toolkits should diverge in parallel, potentially stabilized by physical genomic linkage or co‐localization to reduce recombination. However, population‐level genomic scans evaluating whether host‐associated lineages are maintained by such coupled genetic divergence or if behaviour and physiology can evolve independently remain fundamentally scarce.

In this study, we quantified genomic population differentiation in *Asecodes parviclava* (Hymenoptera: Eulophidae), focusing specifically on venom‐related and chemosensory gene families to elucidate the mechanisms of host‐associated differentiation. This system is uniquely suited for this inquiry due to pronounced interspecific differences in host immunocompetence (where the relative capacity to encapsulate wasp eggs follows the hierarchy *G. pusilla* > *G. tenella* > *G. calmariensis*) and documented phenotypic divergence among wasp populations. Specifically, our previous work showed that: (i) wasps from southern Sweden exhibit higher virulence than those from northern Sweden and (ii) wasp populations associated with the host *G. tenella* exhibit a distinct virulence phenotype compared to those associated with *G. calmariensis* or *G. pusilla* , regardless of geographic origin (Fors et al. 2016). Furthermore, transcriptomic analyses indicate that successful parasitism involves the near‐complete silencing of host immune‐related genes (Yang et al. 2020), suggesting that wasp virulence in *A. parviclava* targets initial phases in the immune cascade.

To investigate the genomic architecture underlying these localized phenotypic shifts, we generated a *de novo* genome assembly and performed whole‐genome re‐sequencing of individual wasps from 17 populations, divided into four groups representing these diverse host‐associations and latitudinal gradients. We then scanned for signatures of selection across both the global genome using targeted candidate gene sets for venom and chemosensory function as an empirical entry point to evaluate two primary expected evolutionary pathways (behavioural host choice vs. physiological host suppression), while acknowledging that non‐candidate loci may also play important roles. Based on the theoretical necessity of coupling preference and performance traits during ecological divergence, we hypothesized that the co‐localization of these two candidate gene sets may serve as a genomic mechanism to stabilize host‐use phenotypes by reducing recombination between recognition and virulence alleles. We further hypothesized that the specific venom loci exhibiting the strongest population‐level differentiation would functionally map to the disruption and silencing of host genes responsible for initiating the early immune response cascade.

## 2 Material and Methods

### 2.1 Study Species

*Asecodes parviclava* is a small koinobiont, endoparasitic wasp (Figure 1) that targets the larvae of three leaf beetle species: *Galerucella calmariensis*, *G. pusilla* and *G. tenella* (Hansson and Hambäck 2013). Wasps overwinter as pupae within host mummies; adults appear in early summer to parasitize larvae, subsequently developing and pupating within the host. Each female may lay multiple eggs in the same host individuals and superparasitism seems common. Consequently, it is not unusual for multiple wasp individuals to hatch from the same mummy (Stenberg and Hambäck 2010).

**FIGURE 1:**
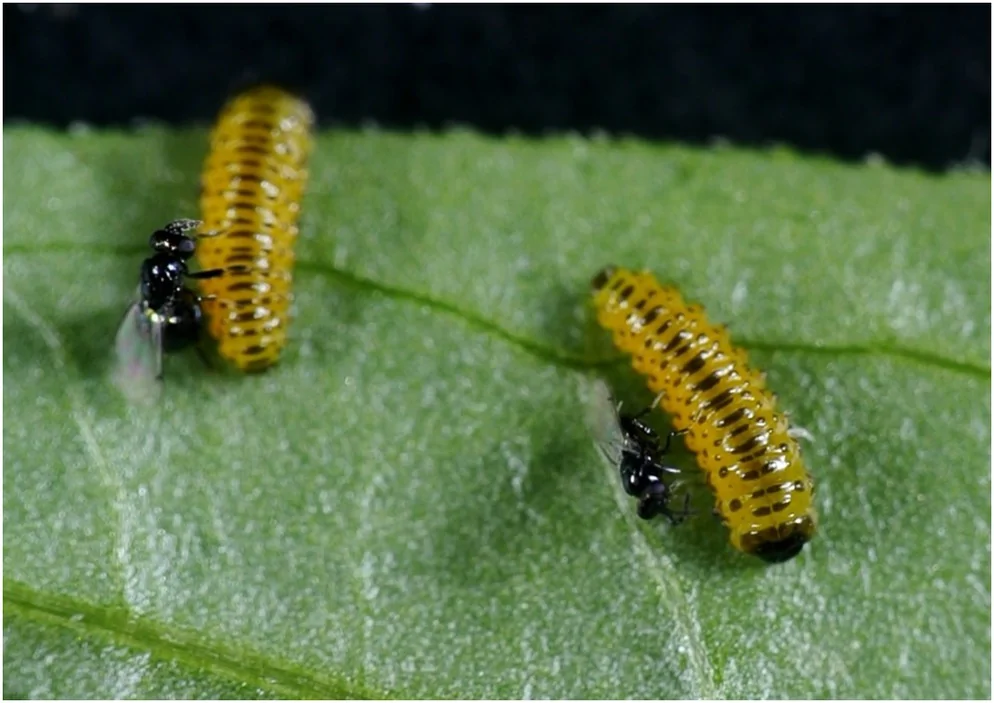
Two *Asecodes parviclava* (Hymenoptera: Eulophidae) females attacking *Galerucella calmariensis* larvae. This beetle species, as well as *G. pusilla* , is monophagous on *Lythrum salicaria* . Wasps commonly move between these two hosts that may occur on the same host plant individual.

Host availability is geographically structured: *G. pusilla* is restricted to southern and central Sweden (limit about 63^o^N), whereas G. *calmariensis* and *G. tenella* occur in both northern and southern sites. Furthermore, hosts occupy distinct ecological niches. *G. calmariensis* and *G. pusilla* , are strictly monophagous on *Lythrum salicaria* (Lythraceae) and frequently co‐occur even on the same plant individual. In contrast, *G. tenella* is oligophagous, mainly using *Filipendula ulmaria* (Rosaceae). The three host species exhibit different immunocompetence. *G. pusilla* mounts a robust encapsulation response against wasp eggs whereas *G. calmariensis* seldom does (Fors et al. 2014). These differences are linked to differences in canonical immune‐related genes and their expression (Yang et al. 2020, 2025). During host finding and selection, wasp females rely on chemosensory cues, including larval and plant volatiles (Fors et al. 2018). Following successfully identification, the female injects both eggs and venom into the host larvae.

We collected *A. parviclava* parasitoids from previous season's parasitized host mummies across Sweden (Table S1), categorized by latitude (hereafter north–south) and host species from which they emerged: North, composed of 3 populations from *G. calmariensis* and 7 populations from *G. tenella* ; South, composed of 3 populations from *G. calmariensis*, 1 population from *G. pusilla* and 3 populations from *G. tenella* (hereafter Gc‐wasps and Gt‐wasps respectively). Adult wasps were collected upon emergence from host mummies. For individuals from northern populations that entered diapause, DNA was extracted directly from wasp pupae. Given the haplodiploid nature of *A. parviclava*, identifying the sex of individuals was critical for downstream bioinformatic processing. For emerged adults, sex was determined via body size and microscopic identification of the ovipositor. For pupal samples, sex was assigned based on genomic heterozygosity levels; males (haploid) exhibit a near‐absence of heterozygosity compared to females (diploid). The validity of this approach was confirmed by comparing heterozygosity levels with morphologically sexed adults.

### 2.2 Genome Assembly and Annotation

For the reference genome, high‐molecular‐weight DNA was extracted from a single haploid male *A. parviclava* using the 10X Genomics ‘DNA Extraction from Single Insects’ protocol. We prioritized long‐fragment DNA (> 20 kb) to facilitate linked‐read sequencing. Sequencing was performed on an Illumina HiSeq X platform (SciLifeLab, Stockholm) to a depth of approximately 100X.

We performed de novo assembly using Supernova v2.0 (Weisenfeld et al. 2017), which utilizes 10X linked‐read information to resolve haplotypes. The primary assembly was further refined by removing redundant haplotigs with purge_dups v1.0.1 (Guan et al. 2020) and improved using the scaffolding tools ARCS v1.0.6 (Yeo et al. 2018) and LINKS v1.8.6 (Warren et al. 2015). Genome completeness was quantitatively assessed using BUSCO v4.1.4 against the endopterygota_odb10 lineage dataset using OrthoDB (Simão et al. 2015).

Genome annotation was executed via the MAKER v3.01.02 pipeline (Holt and Yandell 2011). First, we identified repetitive elements by constructing a species‐specific library with RepeatModeler v1.0.11 (Smit and Hubley 2010), followed by masking with RepeatMasker v3.0.9 (Smit et al. 2010). Protein evidence was pulled from the UniProt Swiss‐Prot database (downloaded on 2019–11; 561,356 proteins) (Bateman et al. 2021) and a curated set of Hymenoptera homologues (uniport request: taxonomy: ‘Hymenoptera [7399]’ existence: ‘Inferred from homology [3]’). We then performed an iterative annotation process: an initial evidence‐based build was used to train ab initio gene predictors, including GeneMark v4.48 (Besemer et al. 2001) and Augustus v3.3.3 (Stanke et al. 2006), which were then integrated into a final evidence‐driven gene build. The quality of the final annotations was validated using the Annotation Edit Distance (AED) metric. Functional descriptions were assigned using BLAST, InterProScan (Jones et al. 2014) and eggNOG v4.5 (Huerta‐Cepas et al. 2016) using Insecta as taxonomic scope.

### 2.3 Population Re‐Sequencing

For the population‐level analysis using a re‐sequencing approach, whole‐body genomic DNA was extracted using the KingFisher Cell and Tissue DNA Kit. We enforced strict quality control, assessed using Nanodrop 8000 and Qubit 3.0 (Thermo Scientific), only including samples with A260/A280 ratios between 1.8 and 2.0. Libraries were prepared using the Rubicon ThruPLEX DNA‐seq kit and sequenced on the Illumina HiSeq X platform (2 × 151 bp) using ‘HiSeq X SBS’ chemistry by SciLifeLab, Sweden.

### 2.4 Population Mapping and SNP Calling

Quality control on the resequencing data was done with FastQC v0.11.5. The reads were mapped against the *A. parviclava* reference genome using BWA‐MEM V0.7.5 (Li and Durbin 2009). The duplicated reads were marked and read group tag added with PICAR v1.118 (https://broadinstitute.github.io/picard/). Before downstream analysis, samples were excluded when ploidy determination was ambiguous or if they exhibited low read depth (< 5×). The resulting data used for analyses included 74 individuals from 4 groups distributed over 17 populations. The SNPs were called using HaplotypeCaller in the Genome Analysis Tool Kit (GATK 4.5.0, McKenna et al. 2010) separately for haploid (ploidy 1) and diploid (ploidy 2) individuals, mapped against the *A. parviclava* reference genome. The resulting gVCF files were soft filtered (*Q* > 20) before joint genotyping with GenotypeGVCFs using default settings. Genotyping was performed by scaffolds and merged using the concat function in bcftools (Danecek et al. 2021). In haploid samples, false heterozygotes—arising from sequencing errors or collapsed repeats—were identified and converted to missing data using a custom script (https://github.com/Jimi92/PopGenScripts/tree/main/MultiPloidy_VCF_tools). We then applied hard filters to the SNPs with bcftools based on GATK best practices (Phred Quality < 30; Quality by Depth < 2.0; Mapping Quality < 40.0; Fisher Strand < 60.0; Strand Odds Ratio > 3.0; Fraction Missing > 0.2). Missing data were imputed using Beagle (Browning and Browning 2007) and removing multiallelic sites. This pipeline resulted in a final high‐quality dataset of 3,051,355 biallelic SNPs.

### 2.5 Genetic Diversity and Population Differentiation

To infer population genetic structure, we excluded rare variants (MAF < 0.05) and performed linkage disequilibrium (LD) pruning on the remaining 1,268,059 SNPs using Plink 2.0 (Chang et al. 2015) with the setting –indep‐pairwise 50 10 0.1. The resulting 153,836 independent SNPs were used in Principal Component Analysis and ancestry cluster estimation using ADMIXTURE v1.3.0 (Alexander et al. 2009). We evaluated a range of ancestral populations (K = 2 to 5) and used the cross‐validation (CV) error to identify the most supported number of ancestral clusters. To further understand gene flow underlying admixture, we reconstructed the evolutionary relationship of populations using TreeMix (Pickrell and Pritchard 2012), comparing scenarios without migration events and those with single or multiple (1–3) events. Migration events in TreeMix are inferred sequentially as directed edges that optimize the model's likelihood by accounting for residual covariance between populations and are therefore agnostic to both temporal and spatial scales.

To identify genomic regions with elevated differentiation between populations, we calculated the fixation index (*F*_ST_) and the genetic diversity (*π*) using non‐overlapping 10 kbp sliding windows across scaffolds > 30 kbp using the Python script popgenWindows.py (https://github.com/simonhmartin/genomics_general). Windows were filtered to require a minimum of 50 genotyped sites. To retain complete local haplotype and frequency data, this analysis was performed using the fully‐filtered variant dataset prior to LD pruning. To calculate F_ST_ across windows, we followed the formulation of Weir and Cockerham (1984) adapted for multi‐locus window‐based analysis. Rather than averaging individual single‐SNP *F*_ST_ values, which can introduce bias from low‐coverage sites, the *F*_ST_ for each 10 kbp window was calculated by summing the components of variance (numerators and denominators) across all valid SNPs within the window before taking the final ratio.

We then performed Gene Ontology (GO) enrichment analysis for biological process categories using the R/Bioconductor package topGO (Alexa and Rahnefuhrer 2010). Gene sets of interest were identified by calculating allele frequency divergence (ΔAF) in four distinct dataset contrasts to identify genes and genomic regions of high differentiation. This approach was chosen in combination with traditional pairwise F_ST_ to provide a more direct, variance‐independent measure of allele frequency shifts at individual loci. To map functional annotations for the enrichment analysis, we parsed the output of eggNOG‐mapper (version 2.1.9). To prevent data inflation from alternative splicing isoforms, individual transcript or protein identifiers (mRNA IDs) were explicitly bridged and collapsed to their respective single parent gene loci, ensuring each locus was counted only once. The background ‘gene universe’ for topGO enrichment testing was strictly restricted to the 16,417 unique gene loci that both contained genotyped SNPs in our dataset and possessed a valid GO annotation.

To identify enriched GO terms, we employed the parent–child algorithm (Grossmann et al. 2007), which reduces false positives by accounting for the hierarchical dependency of the GO graph. Statistical significance was assessed using Fisher's Exact Test. We restricted the analysis to GO terms with at least 5 associated genes (node size = 5). To account for multiple testing across the 50 most significant nodes, we applied a Benjamini‐Hochberg (FDR) adjustment, considering terms significant at an FDR < 10%. Finally, the results were aggregated into a −log10(FDR) matrix to visualize enrichment across all contrasts.

### 2.6 Functional Gene Identification

To further investigate the molecular basis of host‐specific adaptations, we compiled two functional gene sets, targeting venom‐related and chemosensory loci, based on either functional eggNOG‐annotations or previous functional gene characterizations focused on parasitic wasps. For the first set, genes were identified based on eggNOG‐annotations by filtering the global annotation for functional keywords using systematic search strings:

- Chemosensory: odorant, chemosensory, gustatory, olfactory, sensory receptor, pheromone
- Venom/Effector: venom, toxin, protease, peptidase. hydrolase, inhibitor, calreticulin, RhoGAP, SERCA, phosphatase

To assess the genomic architecture of these signals, we identified the physical coordinates of genes in assembly and compared with outlier windows. ‘Adaptive hotspots’ were defined as scaffolds containing clusters of three or more divergent genes from the same functional category.

For the second set, we compiled a list of 107 candidate venom genes from previous functional gene characterizations, venom gland proteomes and gene expression analyses of infected hosts in mainly two parasitoid species ( *Microplitis mediator* , Lin et al. (2019), *Nasonia vitripennis*, de Graaf et al. (2010)) and a list of 91 candidate chemosensory genes from multiple insect groups: *N. vitripennis* , *M. mediator* , *D. melanogaster* and *Apis mellifera* .

The most common protein categories in parasitoid venom include hydrolases and protease inhibitors, though a diverse range of other proteins are also present (Scieuzo et al. 2021). Our candidate list includes 67 putative hydrolases. The most frequent are serine proteases (18), suggested to inhibit melanization of wasp eggs by blocking the host's phenoloxidase cascade (Asgari et al. 2003; Zhang et al. 2004) and metalloproteases (23), which may alter host development and promote parasitoid growth (Lin et al. 2019; Price et al. 2009). Metalloproteases may also directly impair the encapsulation process by suppressing the Toll pathway (Lin et al. 2019). Other hydrolases include proteases, phenoloxidase, acid phosphatase, arylsulphatase and lipases (Scieuzo et al. 2021). Additionally, the list contains 20 putative protease inhibitors, primarily serpins (serine protease inhibitors), which are critical for regulating the prophenoloxidase cascade and preventing melanization (Colinet et al. 2009; Yan et al. 2017). We also identified genes involved in host homeostasis through carbohydrate, DNA, or gluthatione metabolism (de Graaf et al. 2010).

In addition, the list included 19 miscellaneous genes with specialized offensive functions. First, Calreticulin is a calcium‐binding chaperone that acts as an antagonist during the initial encapsulation process by binding to the egg surface and inhibiting hemocyte spreading (L. Wang et al. 2013). Second, RhoGAP domain proteins disrupt the host hemocyte cytoskeleton, destabilize actin to prevent lamellocytes from forming the capsule around wasp eggs (Du et al. 2020; Labrosse et al. 2005). Finally, SERCA (sarco‐endoplasmic reticulum calcium ATPase pump) is an ATPase that hinders the calcium burst needed for early activation of the host immune process (Mortimer et al. 2013).

Chemosensory genes were categorized into three groups. First, odorant receptors (ORs, 51) are expressed in the antennae and maxillary palps to detect volatile signals (Wicher and Miazzi 2021). Second, gustatory receptors (GRs, 15) are involved in taste and pheromone detection across various body parts (Isono and Morita 2010). Third, ionotropic receptors (IRs) have an even more diverse function and are involved in olfactory detection, taste sensation and in detection of environmental cues such as humidity or temperature (Wicher and Miazzi 2021).

To identify orthologs to candidate genes in the *A. parviclava* genome, we used TBLASTN with an E‐ value ≤ 1 × 10^−6^ and a bitscore > 60. Results were cross‐validated against our ab initio evidence‐driven annotation using BLASTP. To reconcile redundant hits across disparate reference taxa, we restricted the dataset to the single top evolutionary match per unique sequence, which yielded 312 unique candidate venom transcripts and 137 unique chemosensory transcripts. These transcript isoforms were subsequently mapped back to their shared structural genomic coordinates. This spatial grouping step successfully collapsed the candidate transcripts down to 190 non‐overlapping, unique venom gene loci and 104 unique chemosensory gene loci (Table S2). Finally, we cross‐referenced these candidate genes with our F_ST_ outlier list to identify potential targets of divergent selection.

## 3 Results

### 3.1 Genome Assessment and Annotation

The de novo assembled *Asecodes parviclava* genome contained 11,196 scaffolds with a N50 = 302,549 bp with a total size of 384 Mbp. We assessed the completeness of this assembly using the BUSCO arthropod database (2124 reference groups). The assembly captured 92.7% of core complete BUSCO groups, consisting of 91.9% (1951) single‐copy orthologs and a remarkably low duplication fraction of 0.8% (17 duplicated groups), alongside 1.8% (39) fragmented and 5.5% (117) missing groups (Table S3). This minimal duplication rate demonstrates a well‐resolved haploid genome assembly. The MAKER annotation pipeline predicted 22,290 protein‐coding gene loci encoding a total of 33,884 proteins (mRNAs), reflecting the retention of alternative splicing isoforms across loci. Out of these models, 18,747 genes (corresponding to 29,692 mRNAs) yielded functional annotation hits and 11,265 genes matched known proteins via BLAST searches, indicating a satisfactory overall quality of the structural and functional annotation.

#### 3.1.1 Genetic Diversity and Population Structure

The genetic diversity level (*π*) varied between populations from 0.00152 (Gc North) to 0.00187 (Gc South). Population structure analyses by PCA reflected the four hypothesized populations (Figure 2), whereas the admixture analysis mainly separated wasps based on host origin (Figure 3). Principal component (PC) 1 separated wasps based on host use (Gt vs. Gc/Gp), PC2 separated Gc‐wasp populations latitudinally (north vs. south) and PC3 separated Gt‐wasp populations latitudinally (north vs. south).

**FIGURE 2:**
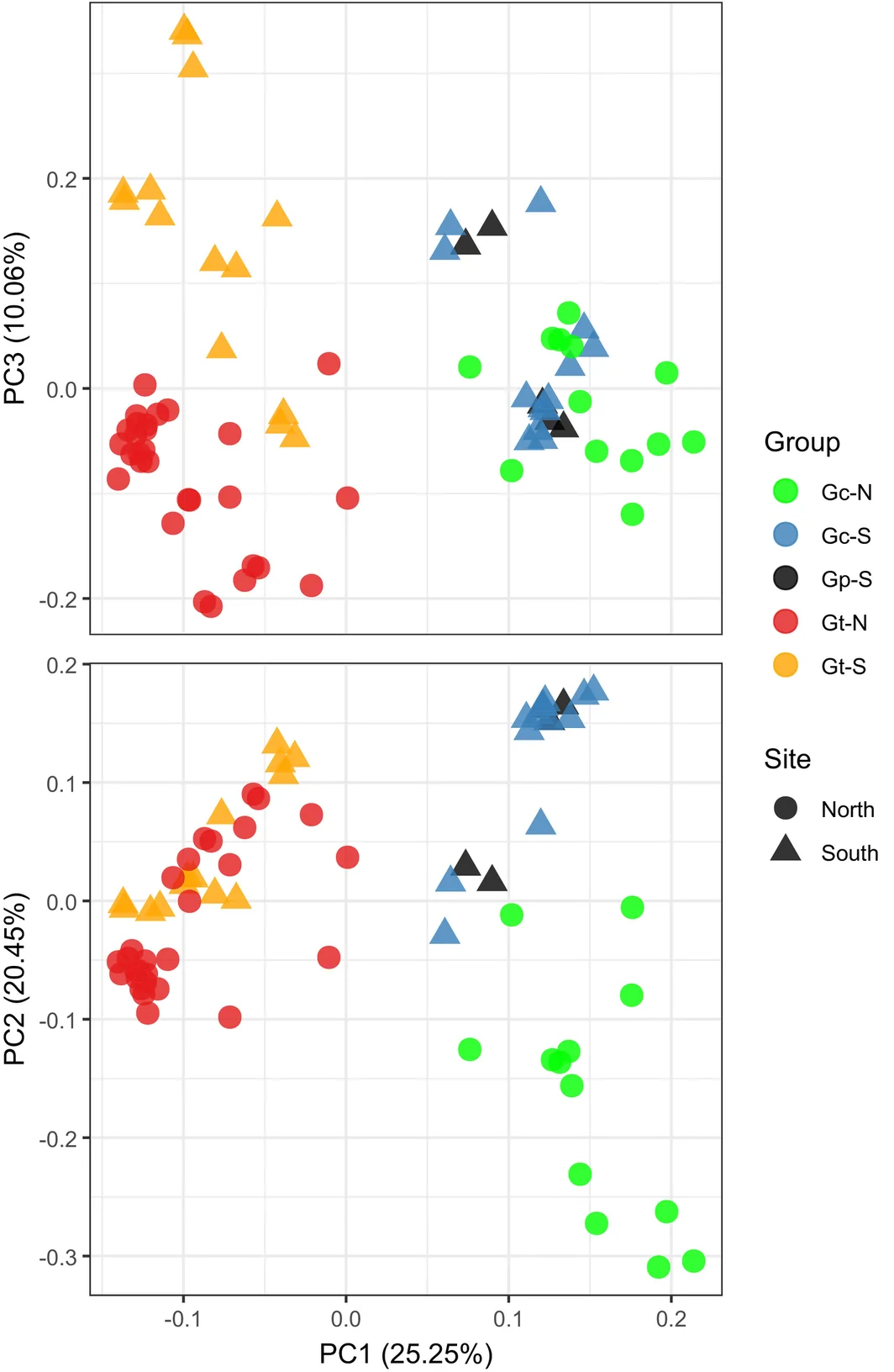
Principal components separating the four populations (Gc‐*N* = *Galerucella calmariensis*‐wasps from northern populations; Gc‐S = *G. calmariensis*‐wasps from southern populations; Gp‐S = *G. pusilla* ‐wasps from southern populations; Gt‐*N* = *G. tenella* ‐wasps from northern populations; Gt‐S = *G. tenella* ‐wasps from southern populations). Principal components were calculated based on independent SNPs (LD‐pruned).

**FIGURE 3:**
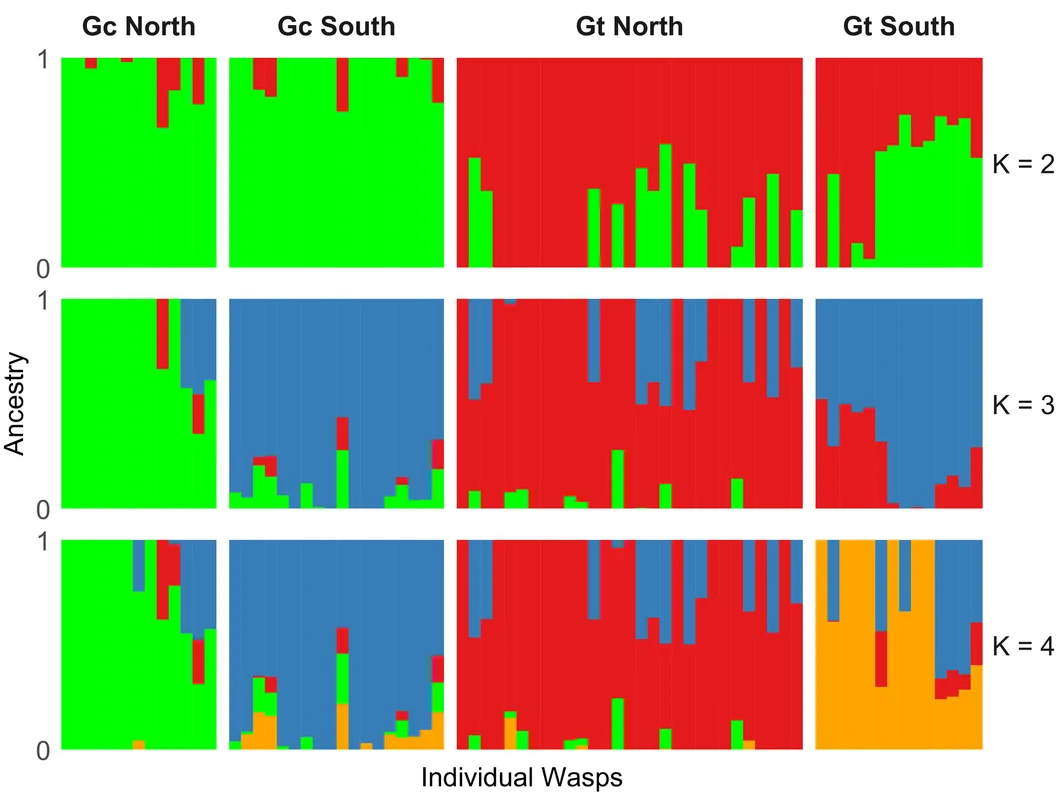
Admixture plot based on 153,836 independent SNPs (LD pruned) for 74 samples (CV‐errors: K2 = 0.80, K3 = 0.82, K4 = 0.85).

The TreeMix analysis suggested that a model with a single migration event (*m* = 1) was superior, explaining 43% of the total genetic variance, which was a substantial improvement over the model with no migration (*m* = 0, 25% total variance). Increasing to two migration events (*m* = 2) resulted in a collapsed model fit (26%), indicating that a single‐event model is the most robust representation of data. The analysis identified a strong, unidirectional migration edge from Gc‐wasps to Gt‐wasps in northern populations (Figure 4), with an estimated migration weight ≈0.5. This high level of admixture in northern populations contrasts with the pattern for southern populations, showing no evidence of interpopulation gene flow. Residual covariance for the *m* = 1 model was negligible across all population pairs, confirming that this asymmetric migration event successfully accounts for the observed genetic relatedness between lineages.

**FIGURE 4:**
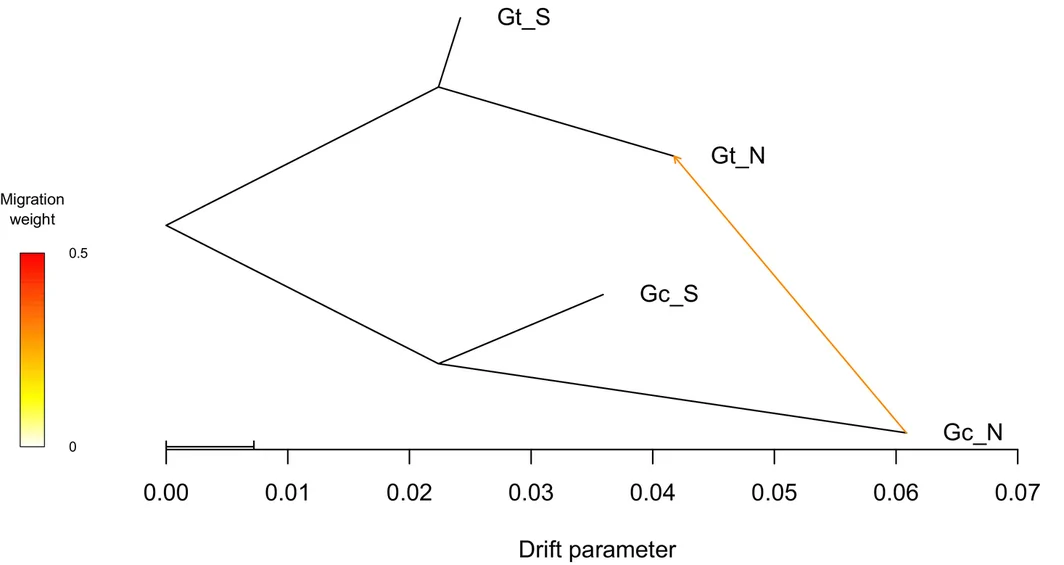
Maximum likelihood phylogeny of parasitic wasp populations (Gc = wasps originating from *Galerucella pusilla/calmariensis*, Gt = wasps originating from *G. tenella* ; S = southern populations; *N* = northern populations), estimated using TreeMix, illustrating the inferred relationship under a model with one migration event (*m* = 1). In Treemix, migration events are inferred sequentially as directed edges that optimize the model's likelihood by accounting for residual covariance between populations. Branch lengths are proportional to the genetic distance between populations. The coloured arrow (colour = the weight of the migration) indicates the predicted migration event.

#### 3.1.2 Identification of *F*_ST_ Outlier SNPs and Candidate Regions of Selection

The overall empirical distribution of window‐based *F*_ST_ estimates across the genome exhibited a distinct bimodal pattern; we consequently applied a filtering threshold of 0.25 to capture the loci comprising this secondary peak of high differentiation. Evaluating pairwise genomic differentiation suggested that one contrast (Gt‐wasps: south vs. north) had a much lower ${\bar{F}}_{\mathrm{ST}}$ (0.032) compared with the other contrast: Gc‐wasp vs. Gt‐wasps in southern sites (${\bar{F}}_{\mathrm{ST}}$= 0.059), Gc‐wasps, south vs. north (${\bar{F}}_{\mathrm{ST}}$= 0.064) and Gt‐wasps vs. Gc‐wasps in northern sites (${\bar{F}}_{\mathrm{ST}}$ = 0.070) (In all contrasts, the majority (82%–96%) of *F*_ST_ < 0.1). This pattern was strengthened in Manhattan plots, showing few outlier‐SNPs in the Gt‐south vs. Gt‐north contrast (Figure 5). Due to the limited differentiation between these populations, we omitted this contrast from further analysis.

**FIGURE 5:**
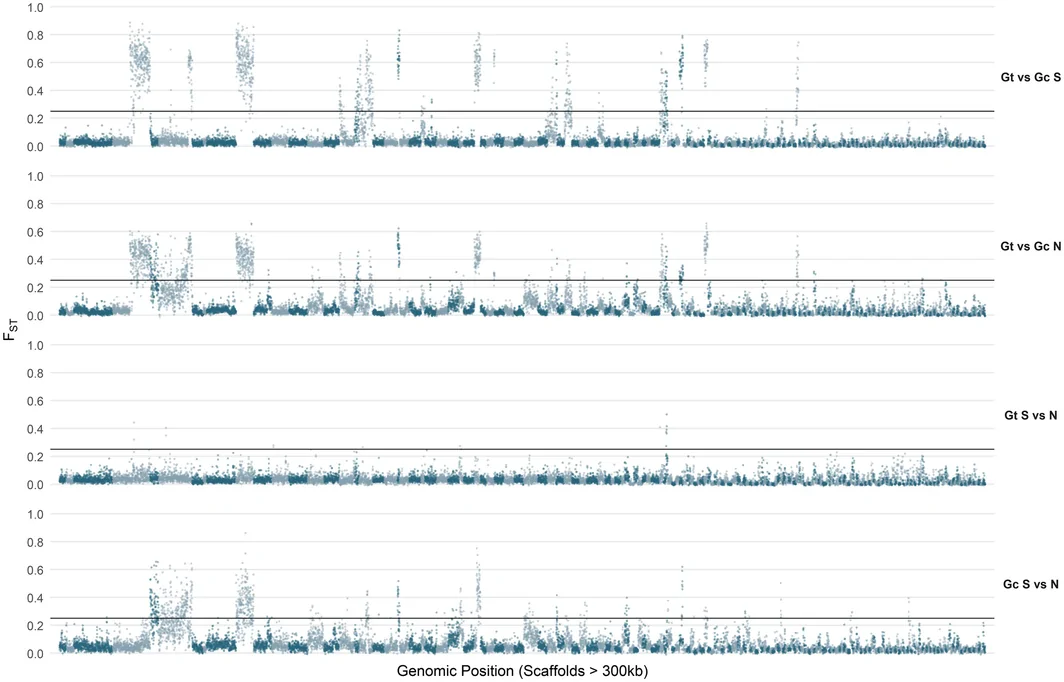
Distribution of Weir & Cockerham's *F*_ST_ values showing population divergence in population contrasts based on host use or latitudinal variation: Gt = wasps originating from *G. tenella* , Gc = wasps originating from *G. calmariensis* or *G. pusilla* , south = southern populations, north = northern populations. Each dot represents average *F*_ST_ in a 10 kb, non‐overlapping window.

##### 3.1.2.1 Broad GO Comparison

The Gene Ontology (GO) enrichment analysis on the unique gene sets, isolated from the top 1% outliers of each population contrast, identified striking functional differences between host‐associated contrasts (Figure S1). First, the enriched GO‐term profiles driving the host‐use contrasts (Gt vs. Gc) were highly conserved across both southern and northern geographic regions, implying that host‐associated selection targets a shared suite of biological processes independent of local latitude. Second, the latitudinal geographic contrast within the Gc population (Gc‐S vs. Gc‐N) yielded a distinct functional signature (Figure S1).

### 3.2 Functional Gene Identification

Our functional gene searches revealed a large number of candidate loci across the *Asecodes parviclava* genome, split between broad automated annotations and targeted homology searches. For the venom‐related candidates, a broad genome‐wide search using eggNOG annotations identified 1303 genes harbouring a total of 34,956 unique physical SNPs. In parallel, our targeted homology search utilizing 107 external reference sequences resolved an expanded set of 190 unique venom gene loci. Within this targeted venom dataset, all 190 genes were polymorphic, containing a total of 20,417 unique physical SNPs within their transcribed boundaries. For the chemosensory candidates, the broad eggNOG annotation pipeline identified 222 genes harbouring a total of 5723 unique physical SNPs. Meanwhile, our targeted sequence‐homology search using 91 reference sequences uncovered 104 unique chemosensory gene loci. Much like the venom candidates, all 104 identified chemosensory genes from our targeted search contained variations, harbouring a total of 6428 unique physical SNPs within their transcribed boundaries. Outlier windows from the F_ST_‐analysis intersected with these functional genes identified multiple venom genes with elevated differentiation between populations. These 29 venom genes were distributed across 9 scaffolds (Table 1, Figures S2–S9). In contrast, only 2 chemosensory genes (*gr28b_2* and *gr28b_3*, both on scaffold 5) were differentiated between populations.

**TABLE 1:** Venom genes intersected with *F*_ST_ outlier windows for the three population contrasts and their position on scaffolds.

Protein	Gene	Scaf	Gt vs. Gc/Gp (S)	Gt vs. Gc/Gp (N)	Gc/Gp S vs. N
Protease Inhibitors/pPO Regulation	SRPN19 (Serpin)	4	✓	✓	
	CLIPA3 (CLIP‐domain SP)	10	✓	✓	✓
	ppaf2_2 (pPO Activating Factor)	6	✓	✓	✓
	SCPEP1 (Serine Carboxypeptidase)	42	✓	✓	
Proteases and Cellular Disruption	KLK7 (Kallikrein)	31	✓*	✓*	✓
	KLK13 (Kallikrein)	4/6	✓	✓	
	CAPN7 (Calpain‐7)	5		✓	✓
	chym2 (Chymotrypsin‐like)	6		✓	✓
	mp1easpe/easpemp1 (Protease)	4/42	✓	✓	
	apeh2 (Acylaminoacyl‐peptidase)	29			✓
Gene Regulation and Epigenetics	ythdf (RNA binding/m6A)	5		✓	✓
	ING3 (NuA4 Complex)	4	✓*	✓*	
	bicra (SWI/SNF Complex)	64	✓		✓
	atad2 (Chromatin modifier)	6		✓	✓
	blm (DNA repair/stability)	4	✓	✓	
Signalling and Metabolism	PPP1CB (Phosphatase)	4	✓*	✓*	
	urh2 (Nucleoside metabolism)	6		✓	✓
	TYSND1 (Peroxisomal protease)	10	✓	✓*	

* Genes with ΔAF > 0.8.

The venom genes with elevated differentiation can be connected to different population contrasts. First, genes with elevated differentiation only between Gt‐wasps and Gc/Gp‐wasps, irrespective of latitude, indicate differentiation depending on host species. These genes are all, except two, co‐located in a 1 Mbp section on scaffold 4 (Figure 6). The six co‐located genes encode a serpin (*SRPN19*), a trypsin (*KLK13*), a protease (*mp1easpe*), a NuA4 complex (*ING3*), a gene coding for DNA repair/stability (*blm*) and a phosphatase (*PPP1CB*). Other genes with elevated differentiation in the host contrast were located on scaffold 10 and 42. Second, there were three genes (CLIPA3, ppaf2_2 and KLK7), located on different scaffolds, that were uniquely differentiated in southern Gc‐wasps and one (*bicra*) with a similar trend. These genes were identified by having elevated differentiation in all contrasts. Finally, five genes, on scaffolds 5 and 6, were uniquely differentiated in northern Gc‐wasps. These genes were identified by having elevated differentiation between hosts in the north as well as between northern and southern Gc‐wasps.

**FIGURE 6:**
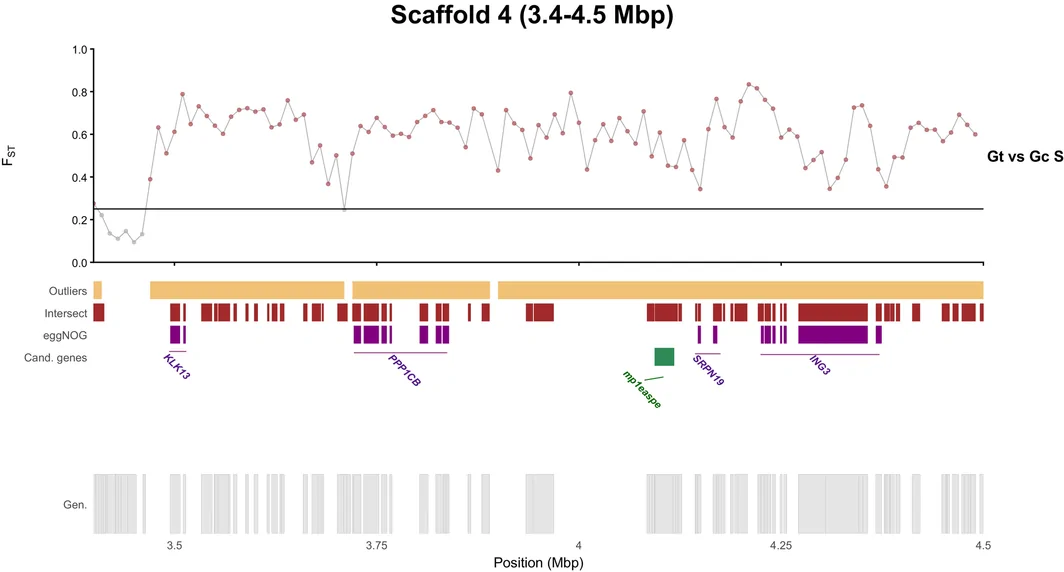
Close‐up image on an outlier gene cluster on scaffold 4 (see also Figure S2), including (from the top) *F*_ST_‐values, outlier regions, intersection between outlier regions and genes, intersections with genes identified through eggNOG, intersections with candidate genes and finally, gene positions.

## 4 Discussion

Our population genomic analysis of *Asecodes parviclava* reveals critical evolutionary processes driving parasitic wasp populations, processes that may reveal mechanisms underlying host‐associated differentiation. First, our analyses suggest three differentiated lineages, corroborating previous phenotypic data. Notably, this differentiation occurs despite the lack of corresponding population structure in their hosts. Second, the most significant genomic divergence exists between wasps hatching from *Galerucella tenella* (Gt‐wasps) and those hatching from *G. calmariensis* or *G. pusilla* (Gc‐wasps). This host‐associated differentiation is consistent across southern and northern localities.

Interestingly, while Gc‐wasps show additional latitudinal differentiation, Gt‐wasp populations remain genomically similar across the ranges studied here. This difference may be linked to the fact that Gc‐wasps differ in their host use between southern (Gc + Gp) and northern (only Gc) populations. We also previously found that southern Gc‐wasp populations possess a higher capacity to overcome host defences than northern ones (Fors et al. 2016). This difference likely stems from the higher selection pressure imposed by the stronger immunocompetence of *G. pusilla* compared with *G. calmariensis* (Fors et al. 2016); the latter is nearly incapable of encapsulating eggs. The intense selection pressure from *G. pusilla* in the south seems to compensate for the higher survival rates on the weaker *G. calmariensis*, as wasps from either host in the south are equally capable of attacking *G. pusilla* . Indeed, cross‐phenotyping showed no functional virulence differences between wasps emerging from *G. calmariensis* and *G. pusilla* in the south and PCA clustering showed Gp‐wasps fully intersecting with Gc‐wasps, justifying their grouping into a single lineage in our analysis. The close physical proximity of these two host species, often co‐occurring on the same plant, likely facilitates gene flow that prevents further host‐associated differentiation between Gc and Gp specialists.

We also found distinct virulence phenotypes in Gt‐wasps compared to the other populations (Fors et al. 2016). Our analysis furthermore identified evolutionary hot‐spots on scaffolds 4–6, where several venom‐related outlier genes are clustered in multiple copies, differently for the host use contrast (Gt‐wasps vs. Gc‐wasps) and the latitudinal contrast (south vs. north for Gc‐wasps). To interpret these patterns and to identify potential key genes explaining the observed phenotypic difference in immune capacity, we first derive some clues from a previous gene expression study of *G. pusilla* and *G. calmariensis*. This study found that 39 canonical immune genes were induced after attack in *G. pusilla* , whereas only one was induced in *G. calmariensis* (Yang et al. 2020). Because no genes were upregulated in *G. calmariensis*, it seems that wasp virulence act to silence genes early in the immune cascade, such as host recognition or signalling genes. Potential candidate genes with these capabilities among the outlier genes include *ING3* and *atad2*. These genes have been suggested to facilitate host suppression by remodelling host chromatin architecture (Dantas et al. 2019; Liakopoulou et al. 2026), thereby epigenetically silencing immune‐related transcription through altered histone modification and chaperone turnover. Another candidate would be *ythdf* which could act through a post‐transcriptional layer of host manipulation. As a primary reader of m^6^A modifications (Wang et al. 2014), *ythdf* may facilitate the selective decay of host immune transcripts, effectively clearing the cytoplasmic mRNA pool before translation into defensive proteins. Furthermore, *PPP1CB*‐like gene products may provide a secondary layer of suppression by dephosphorylating key components of the MAPK or NF‐κB signalling pathways (Bao et al. 2024), ensuring that any recognition signals that bypass epigenetic silencing are halted before reaching the nucleus. Other genes, such as *KLK7*, *CAPN7* and *chym2*, may also be relevant as these could directly interfere with host hemocytes. Parasitoid capacity to interfere with the host immune systems is likely complex, involving multiple interacting gene products. The observed complexes on scaffolds 4, 5 and 6 are therefore key targets for future functional studies. Additional genes of interest may have been overlooked in the analysis due to a fragmented genome, and it is important to note that our gene list, based on homology and gene function, needs to be verified through additional experiments using RNAi or gene editing, similar to recent functional characterizations of parasitoid venom proteins (Huang et al. 2021).

Surprisingly, we found substantial gene flow between wasp populations in northern localities, which was unidirectionally skewed from Gc‐wasps to Gt‐wasps. This observation, however, matches ecological observations: parasitism on *G. tenella* increases in sites where *G. calmariensis* is present (Stenberg et al. 2007). Furthermore, *Filipendula ulmaria* (the Gt host plant) appears more attractive to wasps than *Lythrum salicaria* (the Gc/Gp host plant), likely because *F. ulmaria* is highly aromatic and produces copious nectar (Stenberg et al. 2007). This aromatic difference, causing wasps to switch host plants, occurs primarily in the north because the two plant species often grow side‐by‐side on Baltic Sea shores. In the south, they are ecologically separated: *L. salicaria* occupies wetlands, while *F. ulmaria* grows in drier sites. While this gene flow might be expected to interfere with Gt‐wasp virulence, we hypothesize that hybrid offspring success is highly variable, and only individuals retaining key virulence genes survive the host's defences.

A final surprising result was the relative scarcity of differentiation in chemosensory genes, which stands in sharp contrast to the widespread differentiation in venom‐related genes. While two odorant receptors were significantly differentiated, the overall chemosensory toolkit showed minimal divergence despite known behavioural differences in host attraction (Fors et al. 2018). This discrepancy may indicate that this trait is not genetically fixed (see also Verschut et al. 2017), but rather governed by associative learning or cues acquired upon hatching. If host‐finding is learned rather than ‘hard‐wired,’ it would explain why we see physiological adaptation in venom genes without corresponding genetic changes in the chemosensory system.

In conclusion, our study demonstrates that the diversification of *Asecodes parviclava* is mainly driven by a complex, asymmetrical interplay between host immune landscapes and ecological connectivity. The stark genomic differentiation observed between wasp populations, particularly the profound divergence between Gt‐wasps and Gc‐wasps, highlights the powerful role of host‐specific selection in driving parasitoid evolution. While the host species remain panmictic across wide ranges, the wasps have undergone intense localized selection resulting in significant physiological adaptation, mapping primarily to genomic hot‐spots on scaffolds 4, 5 and 6. These highly differentiated genomic regions harbour a diverse, specialized array of venom‐related genes. Our data indicate that these genes may facilitate host suppression through multi‐layered mechanisms, ranging from the epigenetic silencing of host immune loci and targeted mRNA decay to direct signalling interference. Such complex virulence strategies appear necessary to overcome the highly variable and robust immunocompetence observed across *Galerucella* species. Intriguingly, this physiological adaptation is not mirrored in the wasp's sensory apparatus; the striking lack of differentiation in chemosensory genes suggests that host‐finding may rely more on plastic behavioural traits, such as associative learning, than on fixed genetic changes. This decoupled evolution between the mechanisms used for ‘finding’ and ‘killing’ the host suggests that physiological compatibility, rather than behavioural recognition, is the primary barrier to host‐range expansion in this system. Furthermore, the unidirectional gene flow observed in northern populations underscores how plant community composition and fine‐scale habitat overlap can influence the genetic integrity of specialized parasitoids. Our findings clearly imply that the maintenance of distinct host‐associated lineages is possible even in the face of significant migration, provided that selection on virulence remains sufficiently strong to purge maladapted hybrids. Ultimately, these results provide a concrete genomic framework for future functional studies to dissect the specific roles of these candidate genes in the parasitoid‐host arms race. By integrating genomic, phenotypic and ecological data, we have clarified how a single parasitoid species can successfully navigate the disparate defensive environments provided by closely related hosts.

## Author Contributions

The study was conceived by X.Y. and P.A.H., where T.S. and C.W.W. provided advice on sampling design. Field collections were done by P.A.H. and all genomic work was done by X.Y. Reference genome assembly was done by X.Y. in collaboration with staff at N.B.I.S. and P.A.H. performed the population genomic analyses with advice from C.W.W. and M.F. P.A.H. and X.Y. wrote the first draft of the manuscript and all authors provided comments.

## Funding

This work was supported by the Swedish Research Council (grant no. VR‐2015‐4232, to PAH). The authors acknowledge support from the National Genomics Infrastructure (NGI) through the Science for Life Laboratory, funded by the Knut and Alice Wallenberg Foundation and the Swedish Research Council. The National Bioinformatics Infrastructure Sweden (NBIS) is also acknowledged for bioinformatics support through the PhD advisory program (to XY). Computational analyses were performed on resources provided by the National Academic Infrastructure for Supercomputing in Sweden (NAISS) at the PDC Center for High Performance Computing, KTH Royal Institute of Technology and by the Swedish National Infrastructure for Computing (SNIC) at UPPMAX under project b2013018. MF was supported by funding from SciLifeLab and the Knut and Alice Wallenberg foundation to TS. NAISS and SNIC are funded by the Swedish Research Council through grant agreements No. VR‐2022‐06725 and No. VR‐2018‐05973.

## Disclosure

Benefit Sharing: Field collections have been performed according to Swedish Law, where no special permits are needed to collect invertebrates outside protected areas. All specimens were collected within Sweden and the Nagoya protocol is thereby not relevant.

## Ethics Statement

The authors have nothing to report.

## Conflicts of Interest

The authors declare no conflicts of interest.

## Supporting information


**Figure S1:** Heatmap showing top 30 GO‐terms for three population contrasts with significant outlier SNP regions.
**Figure S2:** *F*_ST_‐values, Outlier regions, Outlier genes (intersect), genes located through EggNOG‐search, candidate genes and all genes for Scaffold 4.
**Figure S3:** *F*_ST_‐values, Outlier regions, Outlier genes (intersect), genes located through EggNOG‐search, candidate genes and all genes for Scaffold 5.
**Figure S4:** *F*_ST_‐values, Outlier regions, Outlier genes (intersect), genes located through EggNOG‐search, candidate genes and all genes for Scaffold 6.
**Figure S5:** *F*_ST_‐values, Outlier regions, Outlier genes (intersect), genes located through EggNOG‐search, candidate genes and all genes for Scaffold 10.
**Figure S6:** *F*_ST_‐values, Outlier regions, Outlier genes (intersect), genes located through EggNOG‐search, candidate genes and all genes for Scaffold 29.
**Figure S7:** *F*_ST_‐values, Outlier regions, Outlier genes (intersect), genes located through EggNOG‐search, candidate genes and all genes for Scaffold 31.
**Figure S8:** *F*_ST_‐values, Outlier regions, Outlier genes (intersect), genes located through EggNOG‐search, candidate genes and all genes for Scaffold 42.
**Figure S9:** *F*_ST_‐values, Outlier regions, Outlier genes (intersect), genes located through EggNOG‐search, candidate genes and all genes for Scaffold 64.
**Table S1:** Summary of included *A. parviclava* populations. In three population, one individual was discarded before analysis due to problem in sex determination or low read depth.
**Table S2:** List of candidate genes (venom related and chemosensory), their position and number of intersecting SNPs. Note: Variant counts (#SNPs) represent total raw variant calls within mapped boundaries. For multi‐copy gene families (e.g., nep2, fbn3), these counts reflect accumulated paralogous sequence variants (PSVs) across duplicated regions, whereas text totals reflect strictly unique genomic physical coordinates (*n* = 20,417 for venom; *n* = 6428 for chemosensory) to eliminate duplication redundancy.
**Table S3:** Summary statistics of *Asecodes parviclava* reference genome, completeness and annotation.

## Data Availability

The data that support the findings of this study are openly available in ENA at https://www.ebi.ac.uk/ena/browser/home, reference number PRJEB114671.
