# Supplementary material for: Host‐Associated Differentiation in a Parasitoid Wasp Connected to Venom‐Related but Not Chemosensory Genes
Source: Mol Ecol. 2026 Oct 12;35(19):e70589. doi: 10.1111/mec.70589 (PMC13655639; doi:10.1111/mec.70589)
Supplement: Supplementary file 1 [file MEC-35-e70589-s001.docx]

**Host-associated differentiation in a parasitoid wasp connected to venom-related but not chemosensory genes**

Xuyue Yang, Marco Fracassetti, Tanja Slotte, Christopher W. Wheat and Peter A. Hambäck

Supplementary information

Table S1. Summary of included *A. parviclava* populations. In three population, one individual was discarded before analysis due to problem in sex determination or low read depth.

| **Population** | **Host origin** | **Coordinates** | **Locality** | **No. of individuals** |
| --- | --- | --- | --- | --- |
| Valasjön | Gp/Gc | 59°3'39"N, 17°16'0"E | South | 5 |
| Näsbo | Gp/Gc | 60°10'56"N, 16°28'46"E | South | 5 |
| Lörudden | Gp/Gc | 62°14'14"N, 17°39'12"E | South | 4 |
| Ornskarpen | Gp/Gc | 61°51'46"N, 17°20'41"E | South | 4 |
| Umeå – 6 | Gc | 63°46'22"N, 20°37'30"E | North | 1 |
| Umeå – 33 | Gc | 63°47'10"N, 20°36'33"E | North | 4 |
| Umeå – 44 | Gc | 63°46'39"N, 20°36'41"E | North | 5 |
| Umeå – 59 | Gc | 63°45'20"N, 20°35'35"E | North | 3 |
| Sala | Gt | 59°55'18"N, 16°34'18"E | South | 5 |
| Bysjön | Gt | 60°26'51"N, 16°27'23"E | South | 5 |
| Sundby | Gt | 59°9'39"N, 16°42'20"E | South | 4 |
| Umeå – 3 | Gt | 63°46'43"N, 20°36'0"E | North | 5/4 |
| Umeå – 6 | Gt | 63°46'22"N, 20°37'30"E | North | 3 |
| Umeå – 15 | Gt | 63°47'9"N, 20°35'53"E | North | 5 |
| Umeå – 16 | Gt | 63°47'3"N, 20°35'43"E | North | 5 |
| Umeå – 37 | Gt | 63°46'15"N, 20°35'23"E | North | 5/4 |
| Umeå – 58 | Gt | 63°46'58"N, 20°34'37"E | North | 5/4 |
| Umeå – 59 | Gt | 63°45'20"N, 20°35'35"E | North | 5 |
| Gp/Gc - S | Gp/Gc |  | South | 18 |
| Gc – N | Gc |  | North | 13 |
| Gt – S | Gt |  | South | 14 |
| Gt – N | Gt |  | North | 32/29 |
| All populations |  |  |  | 77/74 |

Table S2. List of candidate genes (venom related and chemosensory), their position and number of intersecting SNPs. Note: Variant counts (#SNPs) represent total raw variant calls within mapped boundaries. For multi-copy gene families (e.g., nep2, fbn3), these counts reflect accumulated paralogous sequence variants (PSVs) across duplicated regions, whereas text totals reflect strictly unique genomic physical coordinates (n = 20,417 for venom; n = 6,428 for chemosensory) to eliminate duplication redundancy.

| Gene type | Gene ID | Gene name | Scaffold | start | end | Raw SNP count |
| --- | --- | --- | --- | --- | --- | --- |
| Venom | ASPAG00000000020 | nedd4 | 1 | 313768 | 318827 | 37 |
| Chemosensory | ASPAG00000000108 | ir75a_14 | 1 | 2057812 | 2065321 | 138 |
| Venom | ASPAG00000000148 | plca | 1 | 2664152 | 2664861 | 4 |
| Venom | ASPAG00000000192 | snk_6 | 1 | 3029430 | 3030630 | 6 |
| Chemosensory | ASPAG00000000223 | or13a_3 | 1 | 3372760 | 3374228 | 6 |
| Chemosensory | ASPAG00000000224 | gpror4_6 | 1 | 3374673 | 3376230 | 35 |
| Chemosensory | ASPAG00000000227 | gpror4_10 | 1 | 3383335 | 3384684 | 16 |
| Chemosensory | ASPAG00000000228 | or13a_17 | 1 | 3386337 | 3390663 | 208 |
| Chemosensory | ASPAG00000000293 | ir25a | 1 | 3640088 | 3644464 | 270 |
| Chemosensory | ASPAG00000001014 | grik2_3 | 2 | 4588107 | 4603876 | 5760 |
| Venom | ASPAG00000001108 | oplah | 2 | 5742992 | 5752141 | 40 |
| Venom | ASPAG00000001201 | myo9a | 2 | 7184014 | 7195483 | 68 |
| Venom | ASPAG00000001222 | snk_4 | 3 | 95359 | 99708 | 1598 |
| Venom | ASPAG00000001259 | ace_4 | 3 | 422151 | 424552 | 888 |
| Venom | ASPAG00000001289 | prss2 | 3 | 664443 | 665630 | 17 |
| Chemosensory | ASPAG00000001353 | ir93a | 3 | 1460345 | 1463935 | 78 |
| Venom | ASPAG00000001563 | arhgap17 | 3 | 3752156 | 3758866 | 75 |
| Venom | ASPAG00000001661 | lac_2 | 3 | 4894625 | 4926343 | 663 |
| Venom | ASPAG00000001665 | ucp2ucp3 | 3 | 5008573 | 5014646 | 70 |
| Chemosensory | ASPAG00000001694 | gpror4_8 | 3 | 5379833 | 5388033 | 280 |
| Chemosensory | ASPAG00000001697 | gpror4_9 | 3 | 5392373 | 5408230 | 5560 |
| Chemosensory | ASPAG00000001700 | or22c | 3 | 5413906 | 5415598 | 72 |
| Chemosensory | ASPAG00000001702 | or13a_10 | 3 | 5418921 | 5420558 | 44 |
| Chemosensory | ASPAG00000001705 | gpror4_4 | 3 | 5428280 | 5429487 | 68 |
| Chemosensory | ASPAG00000001706 | or13a_8 | 3 | 5429989 | 5431604 | 168 |
| Chemosensory | ASPAG00000001707 | gpror4 | 3 | 5432225 | 5449103 | 1656 |
| Venom | ASPAG00000001725 | btrc | 3 | 5877949 | 5879726 | 64 |
| Venom | ASPAG00000001782 | tasp1 | 4 | 210749 | 212287 | 147 |
| Chemosensory | ASPAG00000001828 | ir75a_20 | 4 | 600168 | 602605 | 1608 |
| Chemosensory | ASPAG00000001829 | ir75a_18 | 4 | 604600 | 609018 | 472 |
| Venom | ASPAG00000002079 | mp1easpe | 4 | 4094731 | 4116569 | 8668 |
| Venom | ASPAG00000002214 | blm | 4 | 5928106 | 5930994 | 33 |
| Venom | ASPAG00000002312 | ythdf | 5 | 699809 | 706712 | 63 |
| Chemosensory | ASPAG00000002316 | gr28b_3 | 5 | 722864 | 724787 | 147 |
| Chemosensory | ASPAG00000002321 | gr28b_2 | 5 | 764510 | 765889 | 162 |
| Venom | ASPAG00000002373 | urh1 | 5 | 1187996 | 1189275 | 29 |
| Chemosensory | ASPAG00000002465 | or1_2 | 5 | 1938338 | 1939745 | 4 |
| Chemosensory | ASPAG00000002508 | ir75a_6 | 5 | 2759281 | 2761798 | 4 |
| Chemosensory | ASPAG00000002611 | or9a | 5 | 3593005 | 3594568 | 36 |
| Chemosensory | ASPAG00000002612 | or22c_5 | 5 | 3595194 | 3596920 | 15 |
| Chemosensory | ASPAG00000002646 | or33a | 5 | 3931257 | 3932828 | 3 |
| Chemosensory | ASPAG00000002649 | or1 | 5 | 3942778 | 3946125 | 63 |
| Chemosensory | ASPAG00000002650 | or94b_3 | 5 | 3946757 | 3948058 | 2 |
| Chemosensory | ASPAG00000002672 | cutc | 5 | 4106991 | 4111035 | 50 |
| Chemosensory | ASPAG00000002675 | or94b_5 | 5 | 4113056 | 4117848 | 64 |
| Chemosensory | ASPAG00000002676 | or94b_2 | 5 | 4118066 | 4120646 | 13 |
| Venom | ASPAG00000002702 | lac12 | 5 | 4271675 | 4276174 | 36 |
| Chemosensory | ASPAG00000002721 | or30a_2 | 5 | 4402450 | 4407593 | 66 |
| Venom | ASPAG00000002840 | asrgl1_2 | 6 | 113065 | 114291 | 78 |
| Venom | ASPAG00000002924 | chia_4 | 6 | 1242322 | 1244415 | 30 |
| Venom | ASPAG00000002956 | urh2 | 6 | 1400512 | 1401803 | 6 |
| Venom | ASPAG00000002957 | urh2_2 | 6 | 1402149 | 1403463 | 15 |
| Venom | ASPAG00000002958 | urh2_3 | 6 | 1404670 | 1405902 | 15 |
| Venom | ASPAG00000003025 | polc | 6 | 2460118 | 2465998 | 98 |
| Chemosensory | ASPAG00000003088 | gr28b_9 | 6 | 3377745 | 3381009 | 504 |
| Chemosensory | ASPAG00000003089 | gr28b | 6 | 3381226 | 3384165 | 1040 |
| Chemosensory | ASPAG00000003091 | gr28b_5 | 6 | 3390612 | 3392018 | 240 |
| Chemosensory | ASPAG00000003093 | gr2a | 6 | 3395231 | 3396617 | 84 |
| Venom | ASPAG00000003121 | atad2 | 6 | 3673826 | 3691988 | 271 |
| Venom | ASPAG00000003180 | chym2 | 6 | 4335178 | 4336063 | 13 |
| Venom | ASPAG00000003231 | ppaf2_2 | 6 | 5114476 | 5116244 | 15 |
| Chemosensory | ASPAG00000003405 | or13a_6 | 7 | 2022103 | 2031723 | 80 |
| Venom | ASPAG00000003830 | serca | 8 | 891923 | 895496 | 20 |
| Chemosensory | ASPAG00000003835 | or63a | 8 | 959345 | 963248 | 14 |
| Venom | ASPAG00000003895 | adamts16 | 8 | 2111720 | 2123739 | 420 |
| Venom | ASPAG00000003987 | cht2 | 8 | 3590284 | 3592869 | 12 |
| Venom | ASPAG00000004084 | mipp1 | 8 | 4699752 | 4701656 | 5 |
| Venom | ASPAG00000004138 | cp1 | 9 | 313312 | 315686 | 506 |
| Venom | ASPAG00000004141 | cp1_2 | 9 | 354907 | 356013 | 264 |
| Venom | ASPAG00000004196 | kyat3 | 9 | 880508 | 882374 | 102 |
| Venom | ASPAG00000004197 | derp3_10 | 9 | 884050 | 884981 | 84 |
| Venom | ASPAG00000004203 | grp | 9 | 973627 | 986284 | 2648 |
| Venom | ASPAG00000004204 | tryp1_10 | 9 | 994111 | 999381 | 972 |
| Venom | ASPAG00000004220 | ppo1ppo2 | 9 | 1202529 | 1205265 | 90 |
| Venom | ASPAG00000004233 | arhgap1 | 9 | 1339481 | 1340969 | 21 |
| Venom | ASPAG00000004259 | nep2_19 | 9 | 1521725 | 1528904 | 918 |
| Venom | ASPAG00000004377 | nep2_16 | 9 | 3519153 | 3522198 | 864 |
| Venom | ASPAG00000004378 | nep2 | 9 | 3525848 | 3533006 | 1596 |
| Venom | ASPAG00000004379 | nep2_4 | 9 | 3535324 | 3537868 | 172 |
| Venom | ASPAG00000004380 | nep2_8 | 9 | 3539158 | 3541813 | 104 |
| Venom | ASPAG00000004407 | ppaf2_10 | 9 | 3697992 | 3699551 | 171 |
| Chemosensory | ASPAG00000004452 | or13a_15 | 9 | 4302750 | 4309033 | 1512 |
| Chemosensory | ASPAG00000004736 | or2aor13a | 11 | 321442 | 324250 | 2256 |
| Venom | ASPAG00000004739 | ten-a | 11 | 334690 | 356158 | 657 |
| Venom | ASPAG00000004759 | gld_9 | 11 | 593479 | 595454 | 47 |
| Venom | ASPAG00000004915 | vgr | 11 | 2396478 | 2398890 | 38 |
| Chemosensory | ASPAG00000004921 | or85c | 11 | 2455641 | 2459966 | 204 |
| Chemosensory | ASPAG00000004940 | or9a_2 | 11 | 2673420 | 2675211 | 90 |
| Venom | ASPAG00000005014 | arsb_5 | 11 | 3235671 | 3238951 | 3 |
| Chemosensory | ASPAG00000005025 | ir25a_3 | 11 | 3337630 | 3341011 | 224 |
| Venom | ASPAG00000005096 | pfas | 11 | 3789397 | 3794300 | 36 |
| Chemosensory | ASPAG00000005139 | or94b_4 | 12 | 175390 | 178816 | 592 |
| Chemosensory | ASPAG00000005140 | or1_3 | 12 | 179243 | 180550 | 274 |
| Chemosensory | ASPAG00000005141 | or94a | 12 | 182320 | 183526 | 76 |
| Chemosensory | ASPAG00000005142 | or94b | 12 | 184152 | 185454 | 90 |
| Chemosensory | ASPAG00000005258 | or10a | 12 | 1528739 | 1530403 | 224 |
| Chemosensory | ASPAG00000005416 | gpror4_5 | 12 | 2416735 | 2418162 | 21 |
| Venom | ASPAG00000005664 | plaa_2 | 13 | 2161014 | 2167096 | 236 |
| Venom | ASPAG00000005665 | plaa | 13 | 2167164 | 2168008 | 6 |
| Chemosensory | ASPAG00000005734 | moody_2 | 13 | 2897426 | 2900260 | 636 |
| Chemosensory | ASPAG00000005825 | orco | 14 | 1097758 | 1105867 | 5040 |
| Venom | ASPAG00000006033 | aceance | 15 | 1569838 | 1574402 | 128 |
| Venom | ASPAG00000006177 | y_10 | 16 | 374936 | 378638 | 101 |
| Venom | ASPAG00000006178 | mrjp2 | 16 | 380276 | 381730 | 16 |
| Venom | ASPAG00000006179 | y_2 | 16 | 384237 | 388222 | 80 |
| Venom | ASPAG00000006275 | ppo1 | 16 | 1235754 | 1241677 | 158 |
| Venom | ASPAG00000006278 | sbspon | 16 | 1251455 | 1259572 | 768 |
| Chemosensory | ASPAG00000006586 | gr28b_8 | 17 | 2214874 | 2216347 | 64 |
| Venom | ASPAG00000006652 | arsb_4 | 18 | 376514 | 381584 | 88 |
| Venom | ASPAG00000006673 | plc_4 | 18 | 709651 | 710478 | 89 |
| Venom | ASPAG00000006705 | plc_7 | 18 | 1147729 | 1148859 | 2 |
| Venom | ASPAG00000006706 | plc | 18 | 1149738 | 1150826 | 1 |
| Venom | ASPAG00000006712 | cg43867 | 18 | 1171325 | 1183980 | 15 |
| Chemosensory | ASPAG00000006746 | or45a | 18 | 1685517 | 1688793 | 18 |
| Chemosensory | ASPAG00000006751 | or67c | 18 | 1697511 | 1702591 | 13 |
| Venom | ASPAG00000006804 | tmc2 | 18 | 2184254 | 2196287 | 23 |
| Venom | ASPAG00000007118 | prep | 20 | 2023259 | 2026227 | 4 |
| Venom | ASPAG00000007158 | nep2_11 | 21 | 45370 | 48187 | 3774 |
| Venom | ASPAG00000007204 | mipp1_2 | 21 | 359273 | 364578 | 140 |
| Venom | ASPAG00000007213 | ece1 | 21 | 459846 | 463833 | 84 |
| Venom | ASPAG00000007229 | adamts1 | 21 | 718096 | 721106 | 268 |
| Chemosensory | ASPAG00000007273 | or1_4 | 21 | 1003072 | 1007485 | 195 |
| Venom | ASPAG00000007369 | tryp3epsilontry | 21 | 1567760 | 1583861 | 315 |
| Venom | ASPAG00000007415 | hmcn1_2 | 21 | 1844157 | 1849804 | 100 |
| Chemosensory | ASPAG00000007441 | tre1 | 21 | 2073671 | 2075499 | 92 |
| Venom | ASPAG00000007608 | hexd | 22 | 1945656 | 1947350 | 6 |
| Venom | ASPAG00000007662 | plc_5 | 23 | 475563 | 476390 | 85 |
| Chemosensory | ASPAG00000007669 | gluria_2 | 23 | 658409 | 673276 | 221 |
| Chemosensory | ASPAG00000007710 | gpror4_7 | 23 | 1292396 | 1294431 | 600 |
| Chemosensory | ASPAG00000007711 | or13a | 23 | 1297009 | 1298655 | 450 |
| Venom | ASPAG00000007872 | eif2a | 24 | 1307970 | 1310525 | 44 |
| Chemosensory | ASPAG00000007981 | or2_6 | 25 | 502080 | 510914 | 2385 |
| Chemosensory | ASPAG00000007982 | or13a_9 | 25 | 511929 | 513679 | 294 |
| Venom | ASPAG00000008258 | mrjp1_4 | 27 | 389457 | 395639 | 136 |
| Venom | ASPAG00000008266 | gld_47 | 27 | 495060 | 497058 | 31 |
| Venom | ASPAG00000008279 | chit1cht2 | 27 | 624334 | 625676 | 40 |
| Venom | ASPAG00000008327 | kyat1_4 | 27 | 1133625 | 1135281 | 180 |
| Chemosensory | ASPAG00000008400 | or2 | 27 | 1806382 | 1812033 | 476 |
| Chemosensory | ASPAG00000008427 | or2_3 | 28 | 70284 | 72531 | 2934 |
| Chemosensory | ASPAG00000008500 | ir75a_11 | 28 | 593663 | 594388 | 18 |
| Venom | ASPAG00000008632 | apeh_2 | 29 | 345922 | 353666 | 102 |
| Venom | ASPAG00000008905 | sod1 | 30 | 1020167 | 1020936 | 42 |
| Chemosensory | ASPAG00000008978 | or30a | 30 | 1409874 | 1411384 | 21 |
| Venom | ASPAG00000009131 | kyat3_8 | 32 | 164078 | 165914 | 100 |
| Venom | ASPAG00000009135 | abr | 32 | 203580 | 208572 | 240 |
| Venom | ASPAG00000009337 | chid1 | 33 | 629550 | 631263 | 80 |
| Venom | ASPAG00000009360 | arhgap26 | 33 | 769410 | 777775 | 270 |
| Chemosensory | ASPAG00000009412 | or13a_2 | 33 | 1065825 | 1067400 | 20 |
| Venom | ASPAG00000009495 | ggt1_3 | 34 | 448946 | 452261 | 138 |
| Venom | ASPAG00000009498 | ggt1_4 | 34 | 477983 | 479961 | 69 |
| Venom | ASPAG00000009499 | ggt1_5 | 34 | 488708 | 491739 | 111 |
| Venom | ASPAG00000009722 | spon2 | 36 | 171903 | 192326 | 1672 |
| Chemosensory | ASPAG00000009975 | or46a | 37 | 1236440 | 1237913 | 70 |
| Chemosensory | ASPAG00000010157 | grid1 | 38 | 2333488 | 2337368 | 6 |
| Chemosensory | ASPAG00000010181 | ir75a_15 | 38 | 2646136 | 2649077 | 6 |
| Chemosensory | ASPAG00000010227 | gr28b_7 | 39 | 673204 | 674907 | 30 |
| Venom | ASPAG00000010299 | modsp_3 | 40 | 15171 | 17896 | 1372 |
| Venom | ASPAG00000010320 | l(3)72ab | 40 | 324181 | 331965 | 180 |
| Venom | ASPAG00000010332 | idgf4 | 40 | 433068 | 435469 | 28 |
| Venom | ASPAG00000010385 | robo2 | 40 | 1156541 | 1163191 | 342 |
| Venom | ASPAG00000010423 | ten-m_2 | 41 | 316989 | 327064 | 238 |
| Venom | ASPAG00000010553 | easpemp1 | 42 | 818941 | 824122 | 342 |
| Venom | ASPAG00000010573 | accoas | 42 | 1200549 | 1209917 | 804 |
| Venom | ASPAG00000010594 | racgap1 | 43 | 64212 | 66938 | 1731 |
| Venom | ASPAG00000010676 | nmtnmt2nmt1 | 44 | 38632 | 53170 | 1825 |
| Venom | ASPAG00000010758 | sodcc.3 | 44 | 894470 | 895649 | 3 |
| Venom | ASPAG00000010822 | aael013712 | 45 | 622079 | 623222 | 45 |
| Venom | ASPAG00000010847 | gld_39 | 45 | 982614 | 991689 | 400 |
| Venom | ASPAG00000010931 | nep1_2 | 46 | 928268 | 931823 | 38 |
| Venom | ASPAG00000011072 | snk_7 | 47 | 985977 | 992889 | 120 |
| Venom | ASPAG00000011075 | nfxl1 | 47 | 1007820 | 1010030 | 11 |
| Venom | ASPAG00000011124 | kyat3_2 | 48 | 703807 | 704927 | 6 |
| Venom | ASPAG00000011314 | aao | 51 | 198261 | 202205 | 108 |
| Venom | ASPAG00000011690 | nrg | 57 | 534559 | 539389 | 222 |
| Chemosensory | ASPAG00000011701 | gpror4_3 | 57 | 628811 | 630470 | 112 |
| Chemosensory | ASPAG00000011702 | or13a_4 | 57 | 631721 | 633111 | 224 |
| Chemosensory | ASPAG00000011703 | or45b | 57 | 635323 | 636725 | 64 |
| Venom | ASPAG00000011784 | arsb | 58 | 495206 | 498781 | 55 |
| Chemosensory | ASPAG00000011792 | gr64f | 58 | 611764 | 617446 | 92 |
| Chemosensory | ASPAG00000011793 | gr64a | 58 | 618210 | 623750 | 286 |
| Chemosensory | ASPAG00000011855 | grik2_4 | 58 | 1127526 | 1132061 | 202 |
| Venom | ASPAG00000011931 | nep2_10 | 60 | 32708 | 36681 | 4711 |
| Venom | ASPAG00000011932 | nep2_13 | 60 | 38139 | 56103 | 84030 |
| Venom | ASPAG00000011936 | nep2_2 | 60 | 77552 | 80179 | 35 |
| Venom | ASPAG00000012009 | kyat3_6 | 61 | 346605 | 348050 | 19 |
| Chemosensory | ASPAG00000012057 | or13a_12 | 63 | 79521 | 80839 | 65 |
| Venom | ASPAG00000012126 | bicra | 64 | 441705 | 449828 | 89 |
| Venom | ASPAG00000012218 | ggt1_2 | 67 | 13210 | 14440 | 62 |
| Venom | ASPAG00000012234 | mrjp2_2 | 67 | 340506 | 342021 | 46 |
| Venom | ASPAG00000012589 | fbn3 | 82 | 289573 | 361621 | 23715 |
| Venom | ASPAG00000012732 | nt5e | 89 | 383514 | 385633 | 132 |
| Chemosensory | ASPAG00000012833 | or13a_18 | 94 | 90415 | 93309 | 528 |
| Venom | ASPAG00000013361 | sodcc.3_2 | 120 | 132289 | 133102 | 33 |
| Venom | ASPAG00000014188 | ir25a_2 | 184 | 136338 | 143613 | 71 |
| Chemosensory | ASPAG00000015076 | ir75a_10 | 265 | 108177 | 114220 | 160 |
| Venom | ASPAG00000015689 | prkdc_2 | 328 | 134476 | 143601 | 110 |
| Venom | ASPAG00000015889 | ctsk | 354 | 125722 | 127697 | 10 |
| Venom | ASPAG00000016448 | k12h4.7_2 | 436 | 39590 | 41557 | 35 |
| Venom | ASPAG00000016449 | prss16 | 436 | 42205 | 44287 | 60 |
| Venom | ASPAG00000016461 | impl2 | 436 | 133125 | 135199 | 27 |
| Chemosensory | ASPAG00000016565 | or2a_3 | 454 | 52192 | 63064 | 1255 |
| Venom | ASPAG00000016572 | rhogapp190_2 | 454 | 117509 | 119118 | 8 |
| Chemosensory | ASPAG00000018875 | gr28b_6 | 993 | 10580 | 11937 | 52 |

Table S3. Summary statistics of *Asecodes parviclava* reference genome, completeness and annotation.

| **Summary of assembly** | |
| --- | --- |
| Sequence number | 11,196 |
| Nucleotide number | 384,195,232 |
| GC content | 38.7 |
| N50 | 302.549 KB |
| **BUSCO results** | |
| Total BUSCO groups searched | 2124 |
| Complete BUSCO hits (%) | 1968/92.7% |
| Complete single hits | 1447/91.9% |
| Duplicated | 17/0.8% |
| Fragment | 39/1.8% |
| Missing | 117/5.5% |
| **Summary of annotation** | |
| Number of protein-coding genes | 22,290 |
| Number of mRNA | 33,884 |
| Average exon length | 275 |
| Average intron length | 471 |
| Average CDS length | 1149 |
| Fraction of the genome covered by genes (intron included) | 14.7 |
| Gene with functional annotation | 18,747 |
| mRNA with functional annotation | 29,692 |
| Number of blast hits | 11,265 |

Figure S1. Heatmap showing top 30 GO-terms for three population contrasts with significant outlier SNP regions.

Fig. S2. Fst-values, Outlier regions, Outlier genes (intersect), genes located through EggNOG-search, candidate genes and all genes for Scaffold 4

Fig. S3. Fst-values, Outlier regions, Outlier genes (intersect), genes located through EggNOG-search, candidate genes and all genes for Scaffold 5

Fig. S4. Fst-values, Outlier regions, Outlier genes (intersect), genes located through EggNOG-search, candidate genes and all genes for Scaffold 6

Fig. S5. Fst-values, Outlier regions, Outlier genes (intersect), genes located through EggNOG-search, candidate genes and all genes for Scaffold 10

Fig. S6. Fst-values, Outlier regions, Outlier genes (intersect), genes located through EggNOG-search, candidate genes and all genes for Scaffold 29

Fig. S7. Fst-values, Outlier regions, Outlier genes (intersect), genes located through EggNOG-search, candidate genes and all genes for Scaffold 31

Fig. S8. Fst-values, Outlier regions, Outlier genes (intersect), genes located through EggNOG-search, candidate genes and all genes for Scaffold 42

Fig. S9. Fst-values, Outlier regions, Outlier genes (intersect), genes located through EggNOG-search, candidate genes and all genes for Scaffold 64
